# Security Techniques for the Electronic Health Records

**DOI:** 10.1007/s10916-017-0778-4

**Published:** 2017-07-21

**Authors:** Clemens Scott Kruse, Brenna Smith, Hannah Vanderlinden, Alexandra Nealand

**Affiliations:** School of Health Administration, Texas State University – San Marcos, 601 University Drive, San Marcos, TX 78666 USA

**Keywords:** Electronic health record (EHR), Firewall, Cryptography, Protected health information (PHI), Security safeguards

## Abstract

The privacy of patients and the security of their information is the most imperative barrier to entry when considering the adoption of electronic health records in the healthcare industry. Considering current legal regulations, this review seeks to analyze and discuss prominent security techniques for healthcare organizations seeking to adopt a secure electronic health records system. Additionally, the researchers sought to establish a foundation for further research for security in the healthcare industry. The researchers utilized the Texas State University Library to gain access to three online databases: PubMed (MEDLINE), CINAHL, and ProQuest Nursing and Allied Health Source. These sources were used to conduct searches on literature concerning security of electronic health records containing several inclusion and exclusion criteria. Researchers collected and analyzed 25 journals and reviews discussing security of electronic health records, 20 of which mentioned specific security methods and techniques. The most frequently mentioned security measures and techniques are categorized into three themes: administrative, physical, and technical safeguards. The sensitive nature of the information contained within electronic health records has prompted the need for advanced security techniques that are able to put these worries at ease. It is imperative for security techniques to cover the vast threats that are present across the three pillars of healthcare.

## Introduction

### Rationale

As defined by the Center of Medicare and Medicaid Services (CMS), “an electronic health record (EHR) is an electronic version of a patient’s medical history, that is maintained by the provider over time, and may include all of the key administrative clinical data relevant to that person’s care under a particular provider, including demographics, progress notes, problems, medications, vital signs, past medical history, immunizations, laboratory data and radiology reports [1].” While it is said that electronic health records are the next step in the evolution of healthcare, the cyber-security methodologies associated with the adoption of EHRs should also be thoroughly understood before moving forward [2]. Due to the sensitive nature of the information stored within EHRs, several security safeguards have been introduced through the Health Insurance Portability and Accountability Act (HIPAA) and the Health Information Technology for Economic and Clinical Health (HITECH) Act.

Confidentiality and security of protected health information (PHI), which is included in a patient’s electronic health record, is addressed in the Health Insurance Portability and Accountability Act (HIPAA). HIPAA was passed by Congress in 1996, however compliance with the sub-rulings regarding security was not required until April 20, 2005 for most covered entities and September 23, 2013 for business associates [3]. The three pillars to securing protected health information outlined by HIPAA are administrative safeguards, physical safeguards, and technical safeguards [4]. These three pillars are also known as the three security safeguard themes for healthcare. These themes range from techniques regarding the location of computers to the usage of firewall software to protect health information. A brief list of safeguards and their definitions is provided in the Appendix.

In 2009, the HITECH Act stressed the significance of reporting data breaches. The HITECH Act maintains specific protocol that is to be followed when reporting data breaches. For example, if an entity encounters a data breach in which the information of 500 or more individuals is compromised, the HITECH Act requires that the entity provide specific details of the breach based upon said protocol [5, 6]. The HITECH Act also mandated Centers for Medicare and Medicaid Services (CMS) recipients to implement and use EHRs by 2015 in order to receive full reimbursements. Incentives were offered to providers who adopted EHRs prior to 2015 and penalties are imposed for those who do not beginning this year. The Office of the National Coordinator (ONC) created the three “meaningful use” stages to be followed by healthcare organizations adopting EHRs. Meaningful use determines the extent to which an entity is utilizing EHRs in comparison to previous patient documentation methods [7]. Currently, the United States healthcare system is in stage two of the meaningful use stages.

There are many aspects of security for technology, which is the reason for HIPAA’s three-tier model of physical, technical, administrative. There are security techniques that fit each of these categories, but there is no panacea of technique to thwart spurious (or accidental) breaches. Technology security officers are trained by many different organizations such as SANS, Microsoft, and the Computer Technology Industry Association. In November 2016, SANS hosted a Healthcare CyberSecurity Summit and Training seminar in Houston, Texas where it provided an overview of the most pressing security issues in healthcare and how to adopt healthy cyber-hygiene habits in the server room. SANS hosts these specialized seminars regularly because the cybersecurity environment is fluid, and because there is no magic combination of security controls and habits that will repel all boarders from key business data. As a result, there is no measuring tool to assess the success of one tool over another: Instead, security professionals balance their security programs with physical, technical, and administrative security controls along with an ever-present eye on the security landscape to observe breaches experienced by others and enact further controls to mitigate the risk of the same breach occurring in their facilities.

### Objective

Through a systematic review of academic journals, this manuscript will discuss the most prominent security techniques that have been identified for healthcare organizations seeking to adopt an electronic health record (EHR) system. The frequency of data breaches in healthcare over the last 2–3 years prompted this research. The reviewers wondered what security measures were discussed as in use in the literature. The intent is to identify those used the most often as an opportunity for industry-wide efforts to secure data for its patients.

## Methods

### Eligibility criteria and information sources

**Fig. 1:**
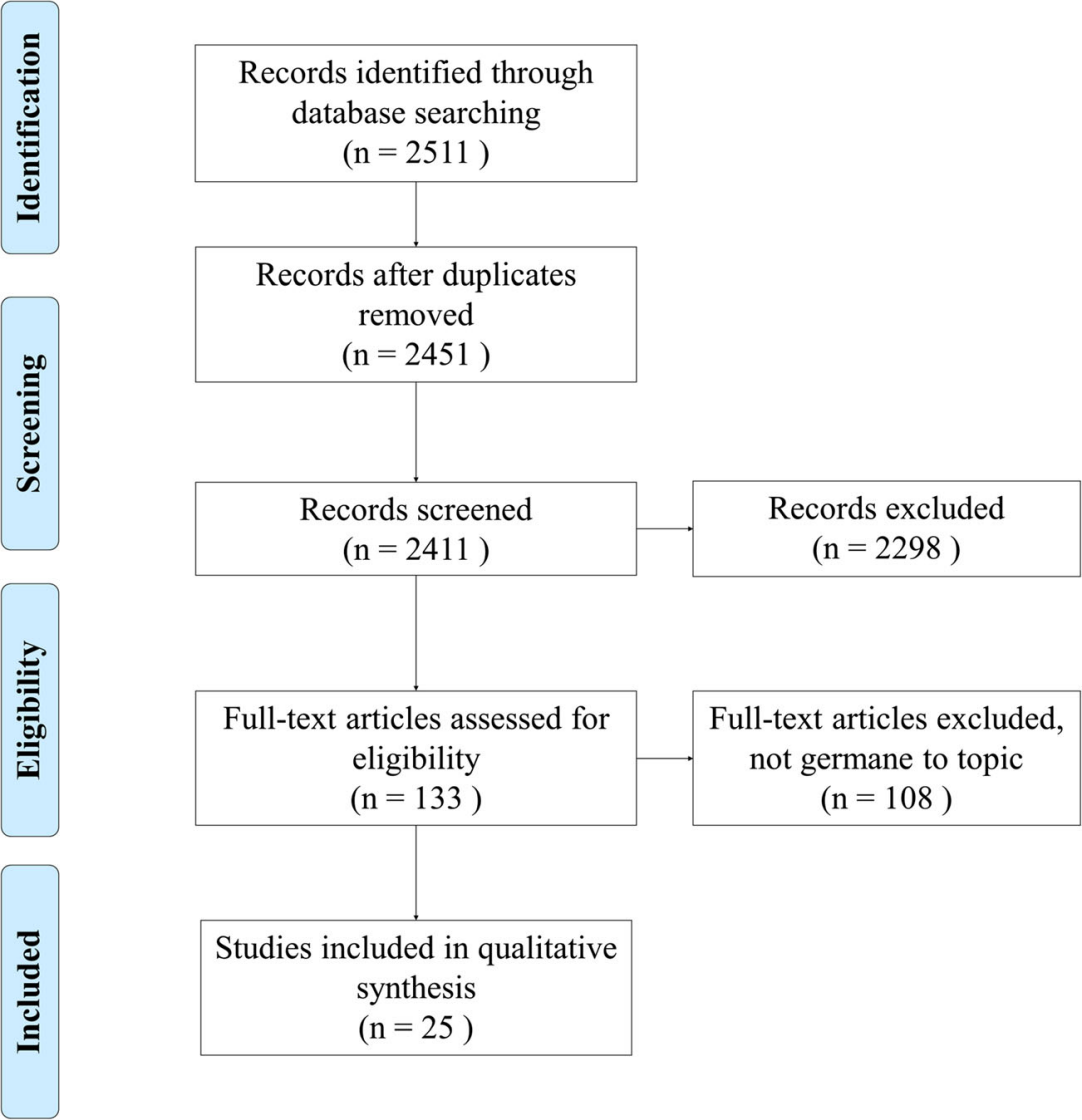
Database research queries

The research gathered for the purposes of this manuscript was obtained from three online databases: PubMed (MEDLINE), CINAHL, and ProQuest Nursing and Allied Health Source. In the initial research conducted on this topic to write the introduction for this work, we found several key terms germane to our objective, and they generated from the Medical Subject Headings (MeSH). The research contained within CINAHL, which stands for cumulative index to nursing and allied health literature, is originally hosted by EBSCO Information Services. The information obtained from PubMed (MEDLINE) originates from the National Center for Biotechnology Information. In PubMed the MeSH automatically links together “electronic health record” and “electronic medical record,” but this link is not established in CINAHL or ProQuest, so both terms were used when querying those databases. The key term of security generated a sufficient level of results for us to feel that it was an exhaustive term. MeSH automatically associated this term with cyber security, computer worms, data protection, data compromising, information protection, data encryption, computer viruses, computer hackers, and data security. The data methodology and criterion used in the researchers’ manuscript is illustrated below in Fig. 1. The three researchers analyzed each research article used in this manuscript.

### Search, study selection, and data collection process

As illustrated above in Fig. 1, the researchers collected 25 relevant research articles through three separate database queries. The researchers used Security AND “Electronic Health Records” as the initial search criteria for all three databases resulting in 1481 results for PubMed, 470 for CINAHL, and 600 for ProQuest. In CINAHL and ProQuest, this search was augmented with “electronic medical record.” In all three databases the choices were screened through a series of criteria. We rejected all articles not published in the English language, the years 2011 through July 2016, in academic journals, and we specifically excluded Medline in CINAHL since it was also included in PubMed. This reduced the number of articles to 133 (41 Pubmed, 34 CINAHL, 58 ProQuest). Each of these articles was reviewed carefully by multiple reviewers for relevancy to our objective. This processed reduced the final group for analysis to 25 (7 from PubMed, 7 from CINAHL, 11 from ProQuest). The time frame for the search criterion was chosen due to the fact electronic health records (EHRs) were not heavily emphasized for implementation until the past few years due to the passage of the Patient Protection and Affordable Care Act (ACA) and “meaningful use” criteria within the Health Information Technology for Economic and Clinical Health (HITECH) Act. While many associate electronic health records with electronic medical records, for the purposes of this manuscript the researchers chose not to include electronic medical records in the initial database search criteria because the researchers were examining security techniques related to fully interoperable information systems. The final group for analysis was 25.

### Summary measures

As reviewers analyzed each article, they looked for common themes (administrative, physical, and technical safeguards) to tie studies together. The reviewers used a series of consensus meetings to refine their search process and discuss the themes. This process enabled the group to progress through the articles expeditiously, and it helped them reach agreement on the summary measures.

### Synthesis of results and additional analysis

Reviewers used a shared Excel spreadsheet to combine and synthesize their observations. This spreadsheet served as the collaboration medium and was the focal point of each consensus meeting. The observations from each reviewer were discussed, which often served as creative motivation to further align the studies in the review. Once a common set of themes were established, it was organized into an affinity matrix for further analysis.

As a group, we decided to analyze each article through the three modalities of security as outlined by HIPAA: Physical, technical, and administrative. We created a column for each of these themes and counted if an article used one or more of them. We also detailed the security techniques mentioned in the article into a summary table.

## Results

### Study selection

Through the database queries, 25 articles were identified for inclusion in this review based upon common security themes and techniques. All 25 research articles were read and analyzed by at least two researchers to ensure their relevance to this manuscript and increase the overall validity of this study.

### Study characteristics and results of individual studies

The security techniques mentioned in the articles were then compiled and listed by article in Table 1. If not already used in the Introduction section, articles are listed in chronological order of publication, the most recent to the oldest.

**Table 1:** Summary of security techniques

Author(s)	Security Techniques
Liu et al. [7]	Physical safeguard: Physical access control to control for theft (locks on laptops); Technical safeguards to prevent electronic breaches (encryption, firewalls).
Amer [8]	Technical safeguard: encryption Administrative safeguards: De-identify samples collected for research
Collier [9]	Technical safeguard: encryption, Administrative safeguards: prevent transfer of patient data off site, anonymize data used for research
Collier [10]	Administrative safeguards: Generators to prevent down time, duplication of all critical hardware, implement comprehensive testing and monitoring strategies
Jannetti [11]	Technical safeguards: firewalls; encryption and decryption; Administrative safeguards: implement comprehensive education and security plans; hire a Chief Information Security Officer (CISO)
Wikina [6]	Administrative safeguards: implement managerial approval paper patient data releases, response training for missing records Phsyical safeguard: security cameras
Ives [4]	Physical safeguard: use locked locations for netework servers Administrative safeguards: game-based security training, establish business-associate agreements with cloud partners Technical safeguards: use role-based authentication and personal-based authentication, use encryption
Hunter [12]	Technical safeguards: Passwords; Antivirus software; Firewalls; Control access; Physical safeguard: Control physical access
Pisto [13]	Technical safeguard: role-based security
Wang et al. [14]	Administrative safeguard: Employing HIPAA consultants
Lemke [15]	Technical safeguards: user ID/passwords; data discard; use short-range wireless (Bluetooth); Privacy enhancing technology (PET) that encrypts fax transmissions Physical safeguard: tamper-proof equipment; Administrative safeguards: policy in place to avoid using wireless devices to store/transmit PHI
Cooper et al. [16]	Administrative safeguard: perform annual risk assessments Technical safeguard: transmit only within guidelines of appropriate standards such as ANSI/AAMI/IEC TIR80001–2-1:2012
Bey et al. [17]	Technical safeguards: Passwords. Anti-virus software. Fire walls. Control access. Physical safeguards: Control physical access. Network access. Unexpected access. Administrative safeguards: Computer habits, mobile devices, security culture.
Chen et al. [18]	Technical safeguard: ID-based authentication scheme
Nikooghadam et al. [19]	Technical safeguard: Mobile agents
Tejero et al. [20]	Technical safeguards: Pseudonymity; encryption; decryption and verification; cryptography (digital signatures, encryption algorithms, digital certificates) Administrative safeguard: digital signatures on all organizational documents
Liu et al. [21]	Technical safeguard: Firewalls
Sittig & Singh [22]	Administrative safeguards: Backups, duplication of critical hardware, train personnel in disaster recovery, reduce interfaces between mission-critical systems and others like pharmacy-management, mandate CPOE for all orders, reduce alert-fatigue Technical safeguard: Implement simple passwords for backup systems
Wickboldt et al. [23]	Phsyical safeguard: Radio Frequency Identification (RFID)
Vockley [24]	Administrative safeguard: perform annual risk assessments Technical safeguard: transmit only within guidelines of appropriate standards such as ANSI/AAMI/IEC TIR80001–2-1:2012
Shank et al. [25]	Technical and administrative safeguard: Digital signatures and associated policies for their use
Lee et al. [26]	Technical safeguard: RBAC Matrix cryptography protocol
Masi et al. [27]	Technical safeguard: Authenticated assertion issuances
Chen et al. [28]	Technical safeguard: Cloud computing
van Allen [29]	Administrative safeguards: training of users to prevent unauthorized disclosure of patient data through inappropriate email, set policies in place regarding social media and social networking, Technical safeguard: access controls to prevent unauthorized access to patient information

### Synthesis of results and additional analysis

**Fig. 2:**
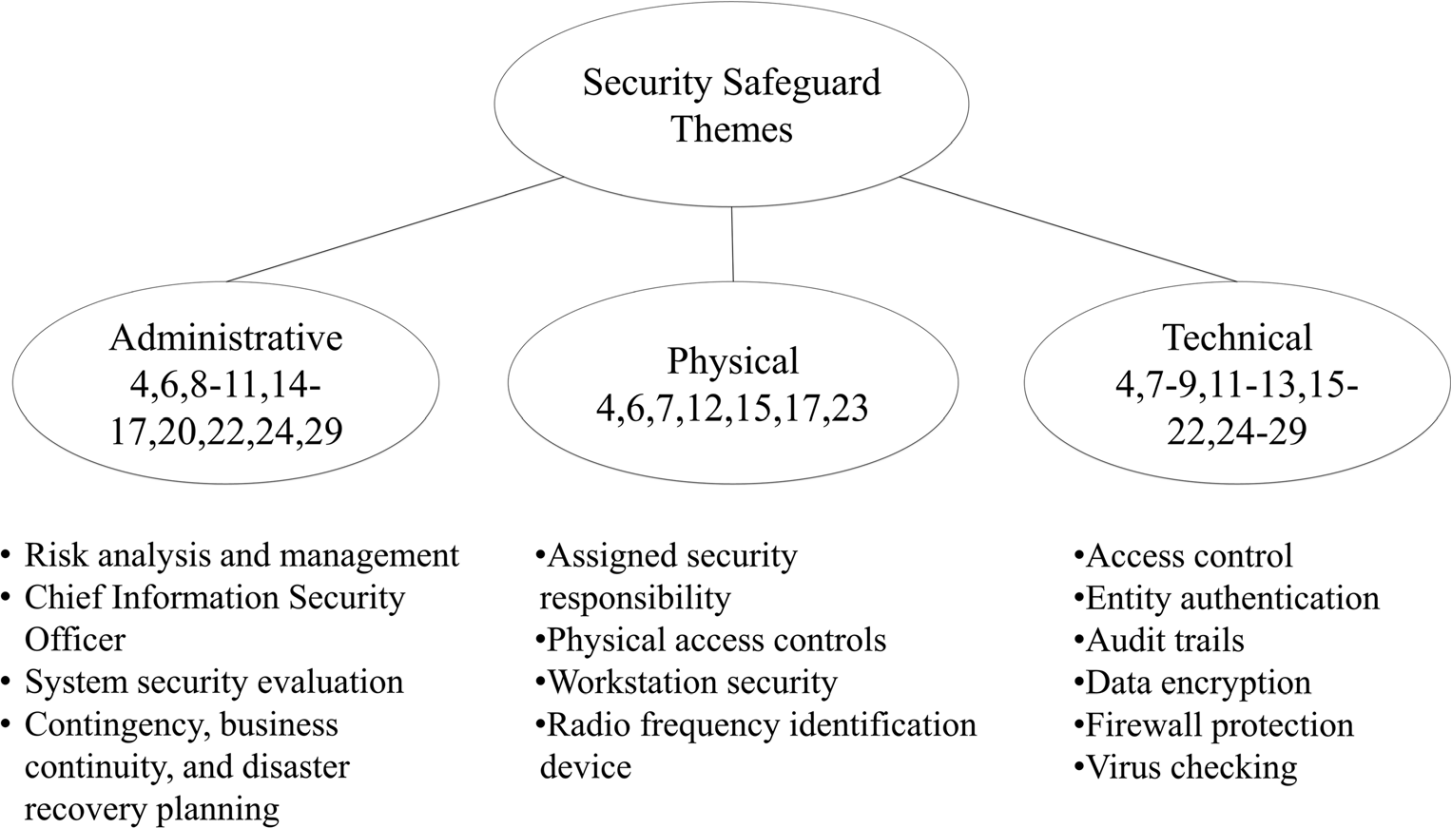
The three themes of security safeguards

Three security-safeguard themes were used to help analyze each article: Physical, technical, and administrative. We identified uses of these themes throughout the research process. Our results are illustrated in Fig. 2. These themes encompass a vast array of security techniques that are implemented by healthcare organizations to further secure protected health information contained within electronic health records. The first theme, administrative safeguards, includes techniques such as conducting audits, assigning a chief information security officer, and designing contingency plans [4, 6, 8–11, 14–17, 20, 22, 24, 29]. Safeguards included in this theme are primarily focused on the compliance of security policies and procedures. The second theme, physical safeguards, includes techniques mentioned in administrative safeguards in addition to focusing on protection of the physical access to protected health information through hardware and software access [4, 6, 7, 12, 15, 17, 23]. Breaches in physical safeguards are the second most common cause of security breaches [7, 30]. Physical safeguards encompass techniques such as assigned security responsibilities, workstation security, and physical access controls [15, 30]. The last theme, technical safeguards, refers to protecting the data and information system that resides within the health organizations’ network [4, 7–9, 11–13, 15–22, 24–29]. This particular theme is crucial for the organization to secure, because most security breaches occur via electronic media, frequently involving laptop computers or portable electronic devices [7, 30]. Security techniques within the final theme include but are not limited to items such as firewalls, virus checking, encryption and decryption, as well as authentication measures [15, 30]. The following section breaks down the themes and discuss individual security techniques identified in the selected research articles.

Currently, privacy and security concerns over protected health information are the largest barrier to electronic health record adoption; therefore, it is imperative for health organizations to identify techniques to secure electronic health records [23]. After analyzing the results, the researchers concluded that the two most frequently discussed security techniques mentioned throughout the selected sample were the use of firewalls and cryptography. Other notable security techniques such as cloud computing, antivirus software, and chief information security officers (CISOs) were also mentioned throughout the readings but implemented based on budgetary schemes and restrictions. The synopsis of the security techniques mentioned Table 1 highlight several interesting points.

The security technique most commonly discussed was the implementation of firewalls to protect the healthcare organizations’ information technology system [9, 11, 12, 15, 21]. While it is known that firewalls can be costly, and vary based upon the size and scope of an organization, they have proven to be very successful in securing an organization’s network and the protected health information that resides on the network. There are several different forms of firewalls that can be implemented both internally and externally to protect the organization from any variety of threats to the information the network possesses. The first type of firewall utilized by an organization is a packet filtering firewall. In a packet filtering firewall system, the organization’s firewall filters internal electronic feeds and prevents outside feeds from entering the organization’s network [7, 30]. This is comparable to when an organization restricts access to specific Internet protocol (IP) addresses. A packet filtering firewall is considered static and the baseline firewall that should be implemented in order to protect the security of electronic health records (EHRs). A second category of firewalls is status inspection firewalls. While this form of firewalls is similar to packet filtering firewalls, they differ in that status inspection firewalls are much more dynamic in the sense that they are able to verify and establish the correlation of incoming electronic feeds with previously filtered electronic feeds [7]. Status inspection firewalls are more complex than the previous category of firewalls and should be implemented in organizations that wish to see the complex correlation of connections of internal and external IP addresses. This type of system takes time and can be expensive, which may not be the best fit for all healthcare organizations seeking to protect the security of EHRs. The third category of firewalls is the application level gateway. This type of firewall acts as a gatekeeper for the organization’s network when scanning the IP web page for any threats prior to forwarding the page on to the end user. In this type of firewall, external network connections are accessed through the gateway in order to prevent external intrusion into the organization’s intranet [7]. Application level gateways have experienced success in securing EHRs because hackers are unable to enter the system directly to obtain protected health information. This category of firewalls tends to be complex and costly for an organization to implement; therefore, a full internal and external analysis of the organization must be done to determine the applicability and viability of the firewall for each specific department as well as the organization as a whole. The last category of firewalls is the network address translator (NAT). The primary function of the NAT is to hide the organization’s intranet IP address from hackers or external users seeking to access the real intranet IP address [7]. This type of firewall creates a barrier between the organizations intranet and the local area network. While network address translators may be costly and complex they are very effective in securing the protected health information within EHRs. While firewalls themselves are considered essential for the security of EHRs, it is also vital that the four phases of the firewall security strategies are followed during implementation. The phases in order are service control, direction control, user control, and behavior control [6]. Overall, it is essential for an organization to complete a full needs assessment, budgetary assessment, and threat assessment, both internal and external to the organization, before adopting any type of firewall. If an organization fails to do so, or fails to complete the four security strategy phases, it could be detrimental to the security of patient’s electronic health records and the organization’s information system as a whole [9, 11, 12, 15, 21].

The use of cryptography has also ensured the security of protected health information in electronic health records systems. Specifically, encryption has enhanced security of EHRs during the exchange of health information. The exchange process of health information has a set specification provided by the meaningful use criteria, which requires the exchange process to be recorded by the organizations when the encryptions are being enabled or inhibited [14, 23]. The Health Insurance Portability and Accountability Act (HIPAA) designed a method for the use of cryptography to ensure security [16]. HIPAA expanded its security and privacy standards when the US Department of Health and Human Services (DHHS) created the Final Rule in 2003 [20]. Under the Final Rule, HIPAA expanded the criteria for organizations when creating, receiving, maintaining, or transmitting protected health information (PHI) [20, 29]. One method specifically mentioned is the use of decryption [6]. For example, decryption ensures the security of EHRs when viewed by patients. Digital signatures are the solution to preventing breaches of PHI when patients view personal information. This method has proven to be a preventative measure of security breaches [11, 24]. Encryption and decryption methods are also successful when used to secure PHI accessed through mobile agents. By securing mobile agents for transmission by patients between facilities, electronic health records are not only more secure, but also more accessible [19]. Another form of cryptography is the usage of usernames and passwords. The utilization of usernames and passwords can ultimately prevent security breaches by simply incorporating personal privacy regarding passwords and requiring users to frequently change personal passwords [15, 18, 30]. The password should not include meaningful names or dates to the individual in an attempt to avoid the likelihood that a hacker could speculate the password. The utilization of usernames and passwords are also a useful security technique for providers in establishing role-based access controls. Role-based access controls restrict information to users based on username and password credentials that are assigned by a system administrator. This security technique protects the information within EHRs from internal breaches or threats [28]. It is also important that the employee remembers to log out of the system after each use to avoid leaving protected health information (PHI) visible to unauthorized personnel [15].

In addition to firewalls and cryptography, other notable security techniques include cloud computing, antivirus software, initial risk assessment programs, radio frequency identification (RFID), and the employment of a chief information security officer. With advancements in technology, cloud computing has become increasingly researched for facilitation and integration in EHR systems. The infrastructures that cloud computing creates allows the electronic transfer and sharing of information through the ‘renting’ of storage, software, and computing power. Through this platform, healthcare organizations are able to cut the costs of adopting an EHR system through shifting ownership and the burden of maintenance, while also integrating cryptography techniques to ensure secure access to the cloud [26]. While cloud computing presents a promising platform, antivirus software remains a consistently used defensive security measure. According to a cyber-security checklist created by The Office of the National Coordinator for Health Information Technology, antivirus software is in the top ten listed methods for avoiding security breaches [12, 28]. In response to the Joint Commission Sentinel Event Alert in 2008, the Food and Drug Administration (FDA), certain manufacturers, and several healthcare organizations convened to create the initial ANSI/AAMI/IEC 80001–1 standard, a technical report that guides specific areas of concern, including security. The ISO/IEC 80001 was created to improve safety, effectiveness, and data system security, in turn recognizing a 10-step process of basic risk management, the initial five specifically outlining risk assessment. These five steps are to: identify initial hazards, identify cause and effect situations from these hazards, estimate the potential harm, estimate the probability of harm, and then evaluate overall risk [16]. As modern technology advances, healthcare organizations are going to continue to be targeted for security breaches. It is imperative that these organizations keep up with new technology and threats, and certain organizations are dedicated to the issue of risk management, including but not limited to: The Clinical Engineering-IT Community (CEIT), the American College of Clinical Engineering (ACCE) and the Healthcare Information and Management Systems Society (HIMSS) [24]. These risk assessment and management steps, as well as the above listed organizations, keep the overall healthcare organization one step ahead in the fortification of patient information within EHRs. A growing number of healthcare facilities are beginning to recognize the security and privacy benefits associated with implementing RFID. Some common RFID techniques include storing data within RFID tags and restricting access to RFID tags to specific devices. These two techniques have enhanced privacy and security through restricting authorized access to a limited number of individuals [25]. Depending on the size and scope of varying healthcare organizations, the utilization of a chief information security officer (CISO) can be helpful, if not essential in order to manage and coordinate all security methods and initiatives used in the fortification of confidential information contained in EHRs [11].

## Discussion

### Summary of evidence

Our review team analyzed 25 articles for security safeguards using the three categories of safeguards in HIPAA: Administrative, physical, and technical. Our team divided the 25 articles among the group in a way that ensured each article was reviewed at least twice. Observations were made on a shared spreadsheet. Details of safeguards mentioned in the literature are listed in Table 1, and they are categorized in Fig. 2. Of the three security safeguard themes, technical safeguards were mentioned 45% (18/40) of all occurrences of safeguards. The next most often mentioned safeguard was Administrative, which was mentioned 17.5% (7/40) of all occurrences of safeguards. Physical security safeguards were only mentioned 12.5% (5/40) of all occurrences of safeguards.

### Limitations

The primary limitation to this study was the failure to specify what types of healthcare organizations were being studied. Narrowing the study to a specific type of healthcare organization, or specifying within the study which security techniques work best for certain facilities, could improve the validity of the study as well as its ability to be generalized to other sectors. Additionally, the researchers failed to consider the various costs of the individual security measures identified. Future research should be sure to identify facility-specific security techniques, in addition to the initial cost, and the implementation and maintenance costs of these security measures.

Another key weakness to this literature review is the lack of litmus test to determine the best program or techniques to prevent data breaches in the healthcare environment. The current HIPAA guidelines set forth compliance measures for physical, technical, and administrative safeguards to provide “adequate” safeguards for confidential data and other key business information. The cyber security professional in healthcare today must keep his/her skills current, much like the medical professional maintaining an annual level of continuing education units (CEUs) to maintain current skills in the field. A technical safeguard of today may not be sufficient when the next version of ransomware surfaces tomorrow; therefore, the security officer in the healthcare facility constantly scans the environment for emerging threats and enacts appropriate safeguards to mitigate the risk to the organization.

### Conclusions

Electronic health records (EHRs) incorporate a vast amount of patient information and diagnostic data, most of which is considered protected health information. With the advancement of technology, the emergence of advanced cyber threats has escalated, which hinders the privacy and security of health information systems such as EHRs.

As mentioned previously, privacy and security concerns present the largest and most important barrier to adopting EHRs. While there are numerous security techniques that could be implemented to prevent unauthorized access to electronic health records, it is difficult to say with confidence what techniques should and should not be used, depending on the size and scope of a healthcare organization. This manuscript identified firewall categories and cryptography methodologies, in addition to a handful of other security techniques. These methods proved to be the most promising and successful techniques for ensuring privacy and security of EHRs, as well as the protected health information contained.

ACA, Patient Protection and Affordable Care Act; ACCE, The American College of Clinical Engineering; CEIT, The Clinical Engineering-IT Community; CINAHL, Cumulative Index to Nursing and Allied Health Literature; CISO, Chief Information Security Officer; CMS, Center of Medicare and Medicaid Services; DHHS, Department of Health and Human Services; EBSCO, Elton B. Stephens Co.; EHR, electronic health records; FDA, Food and Drug Administration; HIMSS, The Healthcare Information and Management Systems Society; HIPAA, Health Insurance Portability and Accountability Act; HIS, Health Information Systems; HITECH, Health Information Technology for Economic and Clinical Health; IP, Internet Protocol; MeSH, Medical Subject Headings; NAT Network address translator; ONC, Office of the National Coordinator; PHI, Protected health information; RFID, Radio Frequency Identification.

## Appendix

Definition of safeguards

1. Access control (technical safeguard) is a technique that prevents or limits access to an electronic resource. The intent behind access control techniques is to limit access to only authorized parties. The healthcare facility collects, stores, and secures patients’ data, which is very sensitive. This safeguard can take the form of *role-based* access control, *attribute-based* access control, and *identity-based* access control. Role-based refers to a person’s role in the healthcare facility. For instance, when a provider begins working at a healthcare facility, he/she has access to patient data, but only the patient data for his/her patients. If this provider also serves on a certain committee in the hospital, then another set of privileges is created to enable access to committee resources. When other data is accessed, a log is created that is periodically audited. When a front-desk clerk begins working in a facility, he/she has no reason to access clinical data, but may need access to the administrative data such as address and phone number, depending on the role that the person plays in the organization. Other names for this are media controls, entity authentication, encryption, firewall, audit trails, virus checking, and packet filtering.
2. Physical access control (physical safeguard) is a technique that prevents or limits physical access to resources. The intent of this control is similar to the technical safeguard: It limits access to only authorized parties. A patient in a facility will not have access to any clinic or ward except the one he/she is seen in. A front-desk clerk in the optometry clinic will not typically need access to the emergency room, so his/her access card will not open those doors. A provider in a facility will not typically need access to the server room, so his/her access card will not unlock those doors. Other names for this are physical security, (some) workstation security, assigned security responsibility, media controls (access cards), and physical access control.
3. Administrative safeguards are techniques that are not entirely technical or physical, but may contain a piece of each. These safeguards typically take the form of policies, practices, and procedures in the facility to regularly check for vulnerabilities and continually improve the security posture of the organization. Other names for this control are risk analysis and management, system security evaluation, personnel chosen for certain roles, contingency, business continuity, and disaster recovery planning.
